# Antibacterial Efficacy and Characterization of Silver Nanoparticles Synthesized via Methanolic Extract of *Fomes fomentarius* L. Fr.

**DOI:** 10.3390/molecules29163961

**Published:** 2024-08-22

**Authors:** Valentina Pavić, Elvira Kovač-Andrić, Ivan Ćorić, Stella Rebić, Zvonimir Užarević, Vlatka Gvozdić

**Affiliations:** 1 Department of Biology, University of Osijek, Cara Hadrijana 8A, 31000 Osijek, Croatia; vpavic@biologija.unios.hr; 2 Department of Chemistry, University of Osijek, Cara Hadrijana 8A, 31000 Osijek, Croatia; eakovac@kemija.unios.hr (E.K.-A.); stella.stella.rebi@gmail.com (S.R.); 3 Department of Laboratory Medicine and Pharmacy, Faculty of Medicine in Osijek, University of Osijek, Josipa Huttlera 4, 31000 Osijek, Croatia; icoric@mefos.hr; 4 Faculty of Education, University of Osijek, Cara Hadrijana 10, 31000 Osijek, Croatia; zuzarevic@foozos.hr

**Keywords:** green synthesis, *Fomes fomentarius*, silver nanoparticles (AgNPs), phytochemical reduction, antibacterial activity, UV-Vis spectrophotometry, FT-IR spectroscopy, PXRD analysis, TEM analysis

## Abstract

Green synthesis employs environmentally friendly, biodegradable substances for the production of nanomaterials. This study aims to develop an innovative method for synthesizing silver nanoparticles (AgNPs) using a methanolic extract of *Fomes fomentarius* L. Fr. as the reducing agent and to assess the potential antibacterial properties of the resulting nanoparticles. The successful synthesis of AgNPs was confirmed through characterization techniques such as UV-visible (UV-Vis) spectrophotometry, Fourier-transform infrared spectroscopy (FT-IR), and powder X-ray diffraction (PXRD). The UV-Vis analysis revealed an absorption peak at 423 nm, while FT-IR identified key phytochemical compounds involved in the reduction process. PXRD analysis indicated a face-centered cubic (fcc) structure with prominent peaks observed at 2θ = 38°, 44.6°, 64.6°, and 78°, confirming the crystalline nature of the AgNPs, with a crystallite diameter of approximately 24 nm, consistent with TEM analysis. The synthesized AgNPs demonstrated significant antibacterial activity, particularly against *S. aureus*, with higher efficacy against gram-positive bacteria.

## 1. Introduction

Due to their specific chemical, physical, and biological properties, nanoparticles, particularly silver nanoparticles (AgNPs), have garnered significant interest in medicine. They are widely used in nanobiotechnological research due to their catalytic and antibacterial activity [1]. They are of great importance due to increased surface relative to volume, altering the mechanical, catalytic, and thermal properties of materials [2]. AgNPs have demonstrated antimicrobial activity against different pathogenic microorganisms [3]. They have numerous applications in medicine, including wound dressing, as drug carriers and AgNPs-modified catheters, and also in diagnosis, detection, and imaging [4,5,6,7,8]. In agriculture, AgNPs are used against plant-pathogenic insects, fungi, bacteria, and viruses and as larvicidal agents [9,10,11,12].

Conventional techniques of nanoparticle synthesis sometimes involve the use of high-energy processes and dangerous chemicals, which can raise concerns about the environment and lead to the production of toxic byproducts. In contrast, by using bio-based materials including plants, microbes, and crop residue as environmentally friendly sources for nanoparticle synthesis, green synthesis methods offer an achievable solution [13]. The main benefits of green synthesis include large-scale manufacturing, an environmentally friendly method, a biological component that serves as the reducing agent, and energy savings as it requires no high pressure or energy [14,15]. It emphasizes the use of reagents that are non-toxic, ensuring that the chemical processes and their products are safe for both humans and the environment, and aiming to create sustainable, energy-efficient processes that preserve natural resources and protect ecosystems [16]. The three primary processes of green synthesis are as follows: choosing a suitable solvent, an environmentally safe reducing agent, and nontoxic compounds that stabilize nanoparticles [1].

Biogenic synthesis of silver nanoparticles (AgNPs) refers to the production of silver nanoparticles using biological organisms or their extracts [17,18,19,20,21]. This method is an eco-friendly and sustainable approach that leverages the natural reducing agents found in plants, fungi, bacteria, algae [22,23,24], or other biological sources such as honey [25] or media like cow, goat, or buffalo urine [26,27,28,29,30] to convert silver ions (Ag^+^) into silver nanoparticles (AgNPs). These sources contain natural compounds like carbohydrates (glucose, lactose, sucrose, fructose, cellulose, starch, and chitosan), proteins (collagen, enzymes, and albumin), amino acids, lipids, nucleic acid (DNA), and phenolic compounds that can act as reducing and stabilizing agents [31,32]. The reduction reaction leads to the formation of silver nanoparticles, which are then stabilized by the biomolecules present in the extract, preventing aggregation and controlling the size and shape of the nanoparticles [33].

Among fungal species, microscopic fungi are the most frequent producers, with about 6400 bioactive compounds exhibiting excellent antibacterial potency and usually low toxicity, while macrofungal species (Basidiomycetes) or mushrooms produce about 2000 active compounds [34]. Hoof fungus (*Fomes fomentarius* L. Fr.) is a woody macrofungus (mushroom) that thrives as a parasite or saprophyte on various deciduous tree species (beech, willow, poplar, linden, birch, beech, oak, maple, etc.), causing heart root of wood [35,36]. For generations, people have used Hoof fungus as a traditional natural cure for a wide range of illnesses and to improve digestion, reduce inflammation, boost the immune system, and heal bleeding wounds [37,38]. Additionally, it was applied to aid in weight loss and prevent stomach and esophageal cancer [38,39]. Numerous physiological activities, such as antibacterial, anti-inflammatory, antidiabetic, antioxidant, anticancer, and antiviral properties, are observed by this mushroom [38,40,41,42,43,44,45,46,47]. Water-soluble melanin–glucan complex (MGC) from *F. fomentarius* L. Fr. effectively inhibited *Candida albicans*, had strong antimicrobial effects on *Helicobacter pylori*, and displayed high anti-HIV-1 activity with low toxicity [43]. *Fomes fomentarius* L. Fr. also showed anti-herpes simplex virus 1 (HSV-1) activities [48]. Inhibitors of plant virus infection with systemic effects known as BAS (Basidiomycete Antiviral Substance) were found in the culture filtrates of *F. fomentarius* L. Fr. and were highly active against tobacco mosaic virus (TMV) without having toxic effects on host plants [49]. The conducted studies indicated the possibility of using exopolysaccharides from the *F. fomentarius* L. Fr. in the treatment of tumors and stomach ulcers since a direct antiproliferative effect in vitro on SGC-7901 human gastric cancer cells was observed [38]. Mushrooms growing on different types of wood, including species of the genus *Fomes*, can yield biomolecules with immunomodulating and antibacterial properties, such as terpenoids and glucans [38,39]. The dried *F. fomentarius* L. Fr. had a β-glucan content of 20.32 ± 0.3 g/100 g, suggesting a potentially rich source of these polysaccharides. For comparison, shiitake mushrooms as the most notable source of β-glucans had a content of 22 g/100 g [50]. Several classes of metabolites were isolated from *F. fomentarius* L. Fr. like esters and lactones, alcohols, ethers, peroxides, sterols, triterpenes, benzofurans, and coumarins [37,51,52,53,54,55]. Zhang and coworkers [56] isolated a total of 18 compounds, triterpenes, and erigosterols from *F. fomentarius* L. Fr. fruiting bodies. Many compounds showed cytotoxicities against six human cancer cell lines and inhibitory activities against nitric oxide (NO) production.

Various macrofungal species have shown great potential in biogenic synthesis, enabling the production of nanoparticles with diverse characteristics [57]. The synthesis of nanoparticles using fungal cell mass or extracellular components has many advantages. Fungal mycelium can withstand pressure, flow, agitation, and other phenomena in bioreactors. They are easy to grow and relatively easy to handle, and the precipitate containing nanoparticles lacks unnecessary components, allowing direct use [58]. To form silver nanoparticles, an extract containing intracellular or extracellular material is mixed with a metal salt solution, with silver nitrate being particularly effective. The reducing agents in the extract can include a combination of biomolecules such as enzymes, proteins, vitamins, flavonoids, amino acids, and alkaloids. Methanolic extracts of different mushrooms are widely recognized for their richness in polyphenols [59], which serve as effective reducing agents in nanoparticle synthesis. Previous research has shown that low molecular weight compounds (phenolic fractions) were efficiently extracted by methanol. Comparative studies have found higher concentrations of phenolic compounds in the methanolic extracts of *C. versicolor* than in its water and ethanol extract [60]. Similar findings were confirmed by Zengin et al. who demonstrated that the reducing capacity of *Asphedoline anatolica* extracts is greater in methanol than in water extracts [61]. Recent publications have confirmed that methanol extract contains significant amounts of polyphenolic compounds that could be active against *H. pylori* [50]. Therefore, in this study, the methanolic extract of *Fomes fomentarius* L. Fr. was used as methanol efficiently extracts low molecular weight phenolic compounds, enhancing the reducing capacity. No studies have been reported on the synthesis of silver nanoparticles using the methanolic extract of *Fomes fomentarius* L. Fr. and their antimicrobial activities. This novel approach leverages the reducing capacity of *F. fomentarius* L. Fr. methanolic extracts, offering a potentially more effective method for nanoparticle synthesis. By utilizing the methanolic extract of *F. fomentarius* L. Fr., this study aims to demonstrate the feasibility of using *F. fomentarius* L. Fr. in the green synthesis of silver nanoparticles, explore the potential antimicrobial applications of the synthesized nanoparticles, and contribute to the development of environmentally friendly methods for producing functional silver nanoparticles with significant biomedical and biotechnological applications.

## 2. Results and Discussion

### 2.1. Characterization of AgNPs Synthesized via F. fomentarius L. Fr. Extract

**Figure 1:**
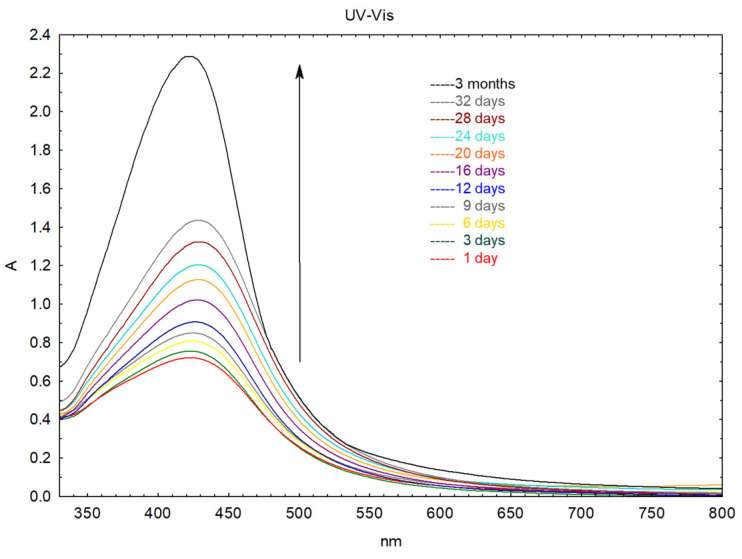
UV-Vis spectra of AgNPs synthesized via *F. fomentarius* L. Fr. extract at different time intervals.

An initial indication that silver nanoparticles were formed by reducing Ag ions after being mixed with *F. fomentarius* L. Fr. extract was the change in color from yellow to brown (Figure S1A,B) [62], due to the excitation of surface plasmon vibrations in the nanoparticles [63,64,65]. AgNPs produced by extracts from *Fomes fomentarius* L. Fr. were examined in a colloidal solution using UV-visible spectroscopy. Figure 1. presents the UV-visible spectrum at specific time points, with the synthesized NPs’ absorbance maxima located at 423 nm. The creation of silver nanoparticles is indicated by the existence of a peak in the 300–500 nm region [64,66]. The UV-Vis spectra offer key information about the dimension, distribution, and form of nanoparticles. It is commonly known that surface plasmon resonances (SPRs), which shift to longer wavelengths with increasing particle size, dominate the optical absorption spectra of metal nanoparticles [67]. This phenomenon occurs when the conduction electrons on the nanoparticle surface resonate with the light wave, resulting in a distinct absorption peak. Furthermore, it is widely recognized that AgNP absorption is mostly influenced by size and shape [68]. The UV-Vis spectra’s lower wavelength maximum shows that the AgNPs that were generated had a smaller diameter; the longer wavelength suggests larger AgNPs [69], while the appearance of two or more maxima at longer wavelengths indicates the presence of large anisotropic nanoparticles [70]. The SPR bands centered between 420 and 430 nm of UV-Vis absorption spectra in Figure 1 align with the peak of silver. Silver nanoparticles exhibiting such SPR bands are expected to be between 10 and 30 nm in size with a spherical shape [71]. Generally speaking, when the nanoparticle’s symmetry rises, the number of SPR peaks drops [72].

Figure 1 exhibited a sharp, narrow, symmetric intense maximum at 423 nm, which indicates the formation of smaller-sized AgNPs. The absorption increased with time, indicating an increase in the number of formed AgNPs. The results of UV-Vis spectroscopy showed that the band located at approximately 423 nm exhibited no shift (bathochromic or hypsochromic) even after three months of measurement. The band maximum value remained constant, without any decrease in absorption maxima or any change in spectral features, which supports the stability of the AgNPs.

**Figure 2:**
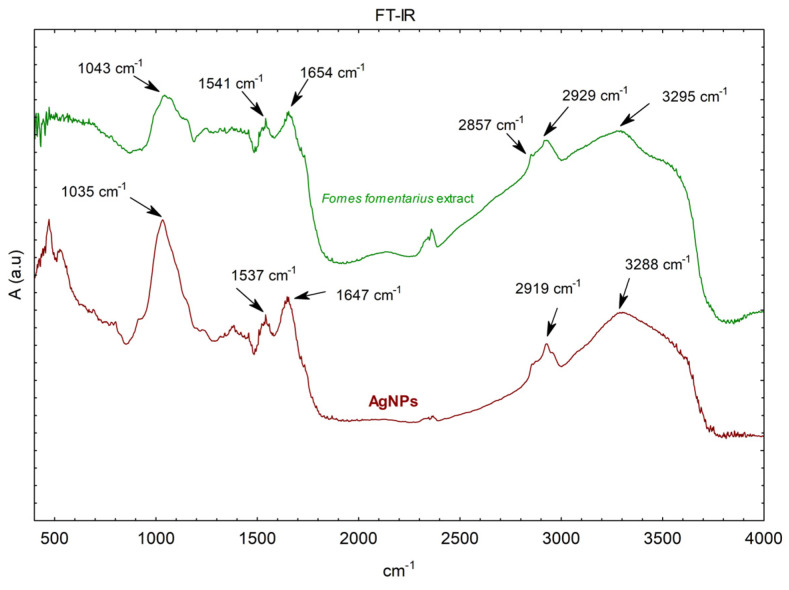
FT-IR spectra of dried *Fomes fomentarius* L. Fr. extract and AgNPs synthesized via *F. fomentarius* L. Fr. extract.

In order to identify the role of the biomolecules responsible for the reduction, FT-IR spectra were recorded in absorption mode between 400 and 4000 cm^−1^. The spectra of dried *F. fomentarius* L. Fr. extract and AgNPs after reaction with AgNO_3_ are represented in Figure 2. The control spectrum showed several peaks indicating the complex nature of *F. fomentarius* L. Fr. extract.

The shifting in wavenumber or changes in peak intensity explains the types of functional groups involved in the binding mechanisms. The FTIR spectrum for *F. fomentarius* L. Fr. dried extract showed absorption bands at 3295, 2929, 1654, 1541 cm^−1^, and 1043 cm^−1^, which were assigned to the O-H linkage of phenolic and hydroxylic groups, C-H stretching vibrations of aliphatic compounds, N-C=O amide I band of proteins, amide II band of proteins, and C-H stretching vibrations of polysaccharides in *F. fomentarius* L. Fr. extract. After reaction with AgNO_3_, there was a shift in the following bands, at 3295 to 3288, 2929 to 2919, 1654 to 1647, 1541 to 1537 cm^−1^, and 1043 to 1035 cm^−1^, indicating that these naturally present organic substances take a key part in stabilization and reduction of Ag^+^. The absorption band at approximately 521 cm^−1^ is not observed in the spectrum of *F. fomentarius* L. Fr. extract. According to Wan Mat Khalir et al., 2020, the absorption peak at approximately 521 cm^−1^ is related to the Ag-O band [73]. According to previous research, the *F. fomentarius* L. Fr. extract is mostly composed of phenolics, carbohydrates, minerals, crude fat, low amounts of proteins, chitin, and paulownin [50,54,74,75]. With β-glucan representing about one-third of the total carbohydrates, along with phenolic compounds like humic acid, lignin, and melanin establishing up the most valuable essential part of the extract [74], the functional groups linked to these biomolecules may contribute to the reduction of Ag^+^ in Ag° [76].

**Figure 3:**
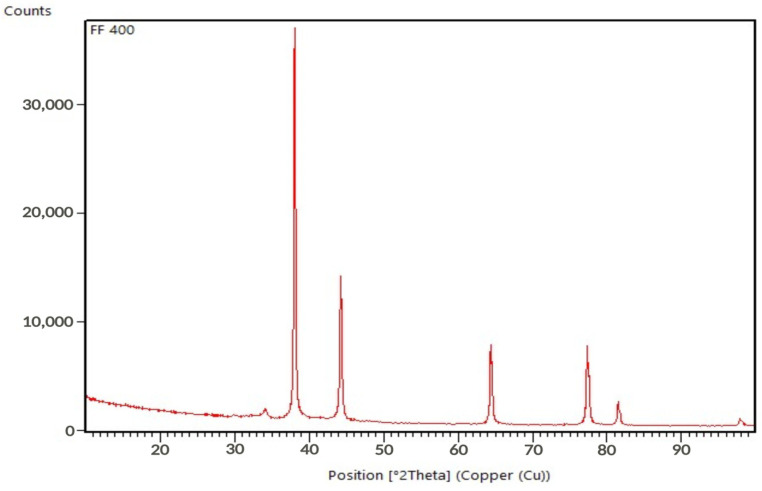
PXRD pattern of AgNPs synthesized via *F. fomentarius* L. Fr. extract.

Further confirmation of the presence of the AgNPs was shown by a PXRD image (Figure 3). The results of PXRD clearly illustrated that synthesized silver nanoparticles are crystalline in nature. The representative PXRD pattern of silver nanoparticles formed after reaction of *F. fomentarius* L. Fr. extract with AgNO_3_ shows the five main peaks observed at 2θ values of 38°, 44.6°, 64.6°, 78°, and 81.5° and are indexed as (111), (200), (220), (311), and (222) crystallographic planes of the face-centered cubic (FCC) structure of silver. The average crystallite size was calculated using Scherrer’s Equation (1):

(1) $$D=\frac{k\lambda }{\beta 0.5cos\theta }$$

where *D* is crystallite size, *λ* is wavelength (1.54056 Å), *β*0.5 (FWHM-full width of half maximum), *k* is the Scherrer constant, and *θ* is the Bragg angle. According to the PXRD results, the synthesized AgNPs attained a size of about 24 nm.

**Figure 4:**
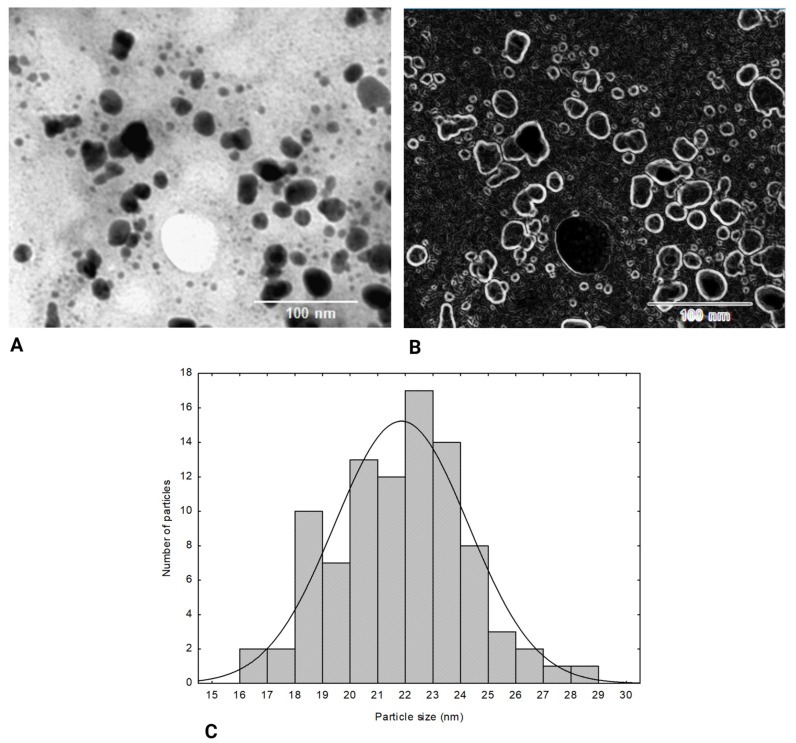
(**A**) TEM image showing the AgNPs synthesized via *F. fomentarius* L. Fr. extract with a size distribution of approximately 23.6 ± 3.5 nm. (**B**) TEM image processed using the “Find Edges” option in the Fiji program (an extension of the ImageJ program). The edge detection enhances the sharpness of the image by defining the particle boundaries more clearly against the background, making the nanoparticles appear more distinct. The scale bar represents 100 nm in A and B images. (**C**) Average particle size of AgNPs synthesized via *F. fomentarius* L. Fr. extract.

The size and morphology of the synthesized AgNPs were further analyzed by TEM analysis, which revealed that the AgNPs were primarily spherical in shape (Figure 4A). Their size ranges between 16 and 29 nm, and the particle diameter distribution histogram indicated that the average particle size was 23.6 ± 3.5 (Figure 4C).

The crystallite size obtained from TEM analysis was consistent with the crystallite size measured by PXRD. As a result, despite a few small agglomerations shown in Figure 4A, the Ag nanoparticles produced using methanolic extracts of *F. fomentarius* L. Fr. are evenly dispersed. The observed clusters may be due to aggregation during sample preparation, which could have also contributed to the reduced clarity and detail in the TEM image. We acknowledge this as a limitation of our analysis and have transparently addressed it in this study. To improve the clarity and boundary definition of the silver nanoparticles, the ‘Find Edges’ option in the Fiji program was applied (Figure 4B). This technique enhances image sharpness by highlighting the edges of the nanoparticles, making it easier to distinguish individual particles from the background. Such enhancement is particularly useful when analyzing the morphology and size distribution of nanoparticles.

This study successfully demonstrated the synthesis of silver nanoparticles (AgNPs) using the methanolic extract of *Fomes fomentarius* L. Fr. However, limitations remain in fully characterizing the crystalline structure. The rationale for selecting UV-visible spectroscopy, FT-IR, and PXRD lies in their proven efficacy in nanoparticle research and wide recognition and consistent use in nanoparticle research. Although they were effective in confirming nanoparticle formation, size, shape, and identifying functional groups, they do not fully capture the electronic states and surface chemistry crucial for understanding interactions with biological systems. UV-visible spectroscopy is essential for detecting nanoparticle formation, as evidenced by the SPR peak at 423 nm, indicating small, spherical AgNPs. This method is also rapid and non-destructive, allowing for the monitoring of AgNP stability over time. FT-IR was employed to identify the specific biomolecules from the *Fomes fomentarius* extract that facilitated the reduction of silver ions and the subsequent stabilization of AgNPs, with shifts in absorption bands indicating interactions between silver ions and functional groups like phenolic and hydroxyl groups, which are known to play active roles in the reduction process. PXRD was used to confirm the crystalline nature and phase purity of the synthesized AgNPs, with diffraction peaks at specific 2θ values indicating a face-centered cubic (fcc) structure, and crystallite size estimated using the Scherrer equation. PXRD is a robust technique for determining the crystal structure and size of nanoparticles, making it a standard choice in nanoparticle characterization.

Given equipment limitations, these key techniques provided substantial insights into the properties of the synthesized AgNPs. However, to further enhance understanding and broaden potential applications, future studies could incorporate additional characterization techniques. Dynamic Light Scattering (DLS) could be used to analyze the hydrodynamic size and zeta potential of the nanoparticles in colloidal suspension, with zeta potential being particularly important for assessing nanoparticle stability in various environments. Energy Dispersive X-ray Spectroscopy (EDX) coupled with TEM could provide elemental analysis and mapping of the nanoparticles, offering more precise information about composition and purity. While TEM was employed in this study, incorporating electron diffraction or X-ray photoelectron spectroscopy (XPS) in future studies would strengthen understanding of the relationship between silver’s oxidation states and the nature of its interaction with biomolecules from the extract, further validating the findings from PXRD and UV-Vis spectroscopy.

### 2.2. Antibacterial Activity of AgNPs Synthesized via F. fomentarius L. Fr. Extract

The results indicated that the initial methanolic *F. fomentarius* L. Fr. extract exhibited significant antibacterial activity, showing the highest efficacy against *E. coli*, with an MIC value of 2.63 mg mL^−1^, less potency against *S. aureus*, with an MIC value of 10.41 mg mL^−1^, and the least activity against *B. subtilis* and *P. aeruginosa,* both with an MIC value of 20.83 mg mL^−1^ (Table 1).

These findings are in contrast with previous studies. For instance, Kolundžić et al. (2016) [50] reported an MIC value of 125 µg mL^−1^ against *E. coli*, *S. aureus*, *B. subtilis*, and *P. aeruginosa*, indicating broad-spectrum activity with a higher potency than that observed in our study. Dundar et al. (2016) [77] found that their extract did not exhibit activity against *B. subtilis* and *E. coli*, but it showed a 4 mm inhibition zone against *S. aureus* and an 8 mm inhibition zone against *P. aeruginosa*. This suggests that the methanolic *F. fomentarius* L. Fr. extract used in our study may possess a unique composition or higher concentration of active compounds that confer better efficacy, especially against *E. coli*. On the other hand, İrez et al. (2021) [78] reported that their methanolic extract was most effective against *B. subtilis* and *E. coli*, with a remarkably low MIC value of 2.5 µg mL^−1^, which is significantly more potent than our findings. The variations in antibacterial activity across these studies may be attributed to differences in extraction methods or the source and concentration of bioactive compounds.

However, the silver nanoparticles demonstrated independent antibacterial properties. The AgNPs synthesized using the methanolic extract of *F. fomentarius* L. Fr. demonstrated antibacterial activities against *E. coli*, *P. aeruginosa*, *B. subtilis*, and *S. aureus* (Table 1). The AgNPs were most active against *S. aureus*, with an MIC value of 6.34 µg mL^−1^. In addition, AgNPs were active against *E. coli*, *P. aeruginosa*, and *B. subtilis*, with no differences in activity, each having an MIC value of 12.69 µg mL^−1^.

These results support several earlier studies that have shown the antibacterial efficacy of AgNPs biosynthesized utilizing mushrooms. Using water extracts of *Pleurotus giganteus*, a wild edible mushroom, Debnath et al. (2019) [79] synthesized AgNPs with MIC values of 12, 10, 14, and 15 μg/mL against *E. coli*, *P. aeruginosa*, *B. subtilis*, and *S. aureus*, respectively.

Zhang et al. (2020) [80] synthesized silver nanoparticles (AgNPs) using *Flammulina velutipes* mushroom extract. TEM analysis showed monodispersed spherical AgNPs averaging 22 nm, while X-ray diffraction confirmed their face-centered cubic crystalline structure and showed excellent stability and antibacterial efficacy against six aquatic pathogens. Extract from reishi mushrooms *Ganoderma lucidum* was used to synthesize AgNPs by Aygün et al. (2020) [81]. TEM images revealed spherical AgNPs with diameters of 15–22 nm, and a face-centered cubic structure was verified by XRD. The AgNPs showed significant antibacterial action against gram-positive (*S. aureus*, *E. hirae*, *B. cereus*) and gram-negative (*E. coli*, *P. aeruginosa*, *L. pneumophila* subsp. *pneumophila*) bacteria. Additionally, they demonstrated a significant level of antifungal activity against the *C. albicans* fungus. A variety of different studies have been carried out regarding the production of silver nanoparticles from fungal organisms, including *Verticillium* sp., *Fusarium semitectum*, *Penicillium* sp., *Aspergillus niger*, *A. terreus,* etc. [82,83,84,85,86]. To our knowledge, this is the first report of the effective synthesis of mushroom-mediated AgNPs using a methanolic extract of *Fomes fomentarius* L. Fr.

Numerous researchers have documented AgNPs’ antimicrobial activity. Nonetheless, a significant degree of variance was seen in the MIC values from the earlier investigations. Because diverse approaches have been used by the researchers and there are different methods for determining the antibacterial activity of AgNPs, it is challenging to compare the results. When compared to the results from the literature, the AgNPs synthesized in this study using *F. fomentarius* L. Fr. extract show significant potency. For instance, the MIC value of AgNPs against *E. coli* was 65 µg mL^−1^, against *P. aeruginosa*, it was 75 µg mL^−1^, against *B. subtilis*, it was 180 µg mL^−1^, and against *S. aureus*, it was 85 µg mL^−1^, as demonstrated by Devanesan and Alsahi (2021) [87]. While several studies have previously reported on the antimicrobial activity and potential action mechanisms of silver nanoparticles (AgNPs), our findings represent exceptionally strong antistaphylococcal activity exhibited by AgNPs. It has been previously highlighted that the relatively small size of the spherical Ag nanoparticles is conducive to determining their considerable antimicrobial activity [88]. Various antibacterial action mechanisms are thought to be useful in restricting bacterial growth. First, AgNP surfaces go through a number of processes, including aggregation, disintegration, photochemical reactions, and the Ag ions’ discharge. Second, AgNPs engage with the phosphorus moiety in bacterial DNA, which inactivates replication and ultimately causes bacterial cell death by impeding bacterial growth. This interaction also occurs with bacterial cell walls, impairing the normal function of bacterial proteins. However, AgNPs’ physical and chemical characteristics, size, shape, and ligand chemistry, in addition to the medium, bacterial habitat, and concentration, all have an impact on their antibacterial activity [89,90].

Although this study demonstrated the significant antibacterial activity of AgNPs synthesized using the methanolic extract of *Fomes fomentarius* L. Fr., it did not investigate the specific molecular mechanisms underlying their interaction with bacterial cells or the specific mechanisms of action, such as the generation of reactive oxygen species [91] or the potential disruption of bacterial biofilms [92]. Future research using advanced techniques like proteomics, transcriptomics, and metabolomics could provide deeper insights into how AgNPs disrupt bacterial cell function at the molecular level [93,94]. Additionally, employing electron microscopy, fluorescence microscopy, and other imaging techniques could visualize AgNP interactions with bacterial cells and biofilms in real-time. In vivo studies in animal models could be conducted to assess the biodistribution, pharmacokinetics, and long-term effects of AgNPs. Furthermore, future studies should evaluate the ecological impact of AgNP release into the environment, focusing on effects on non-target organisms and ecosystems. Developing strategies to minimize environmental contamination, such as recycling or safe disposal of AgNPs, is crucial for sustainable development.

*Fomes fomentarius* L. Fr. has garnered our attention for green nanoparticle synthesis due to its unique properties and potential advantages, including sustainability, cost-effectiveness, biocompatibility, and efficiency. As a widely available, renewable, and biodegradable resource, it represents an environmentally friendly option for nanoparticle production. This mushroom contains natural compounds like polysaccharides, polyphenols, and proteins, which can be utilized as stabilizing and reducing agents in the synthesis of silver nanoparticles. The application prospects of AgNPs synthesized using *F. fomentarius* are substantial and promising, particularly in several industrial and medical domains. In the field of catalysis, these AgNPs could serve as highly efficient catalysts for various chemical reactions due to their large surface area and enhanced reactivity, potentially revolutionizing processes in the chemical industry. In water purification, AgNPs produced through this green synthesis method could be employed to remove contaminants, including pathogens, heavy metals, and organic pollutants, thereby contributing to safer and cleaner water sources [95,96]. Medically, the biocompatibility [97] and antimicrobial properties of these AgNPs provide opportunities for applications in wound healing, biomarker detection, and targeted drug delivery systems. Their potential to be used in diagnostic tools, such as biosensors for detecting specific biomarkers [98], could lead to advancements in early disease detection and personalized medicine. Furthermore, the ability of these nanoparticles to be customized for targeted drug delivery presents options in cancer therapy [99], where they could deliver therapeutic agents directly to tumor cells, minimizing side effects on healthy tissues.

Given the growing concern about environmental sustainability and the need for greener technologies, the use of *F. fomentarius* L. Fr. in AgNP synthesis aligns with the increasing global focus on more sustainable practices. The ability to produce AgNPs with specific properties for various applications highlights the agility and potential of this method. Future research focused on optimizing the synthesis process, expanding production, and rigorously evaluating the environmental and health impacts will be crucial in transitioning these promising applications from the laboratory to industrial and clinical settings.

## 3. Materials and Methods

### 3.1. Collection of Fomes fomentarius L. Fr.

**Figure 5:**
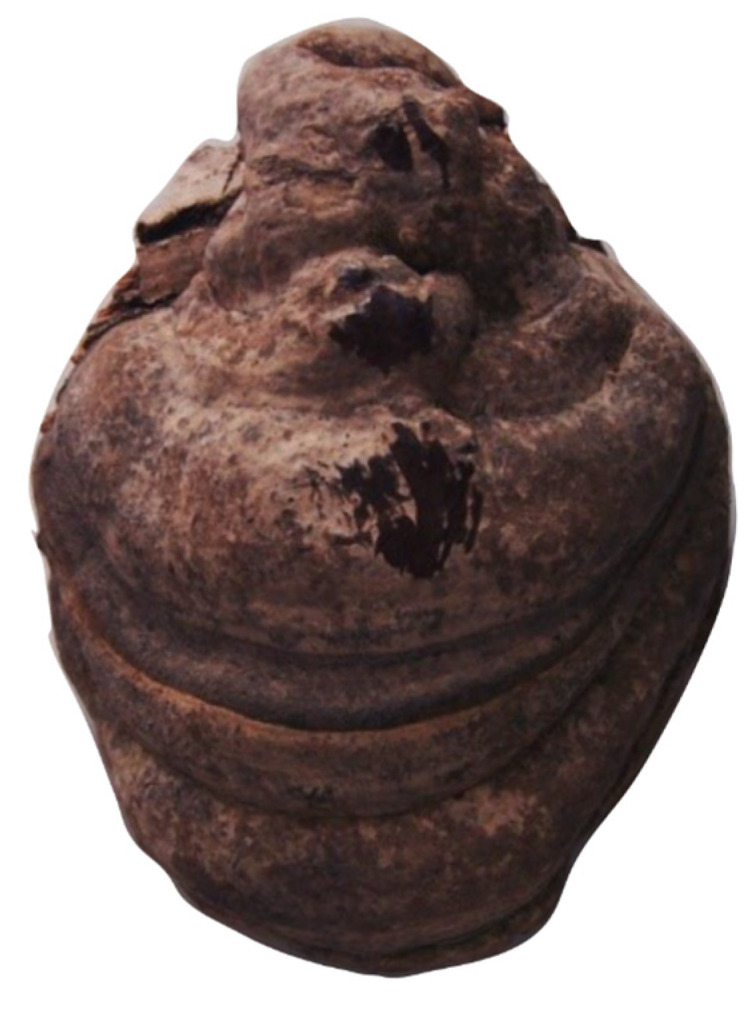
*Fomes fomentarius* L. Fr.

*Fomes fomentarius* (L.) Fr. was collected in the Osijek-Baranja County area (45.61731317289811, 18.50216958907446) from the beech tree *Fagus sylvatica* (L.) (Figure 5). Samples were transported to the laboratory for an extensive morphometric analysis after being appropriately labeled and assigned a voucher number. Using standard keys field guides and manuals, specimens were identified by careful study of characteristics. After being dried, the specimen was deposited at the Department of Chemistry, University of Osijek, Croatia.

### 3.2. Preparation of AgNPs

Dried *F. fomentarius* L. Fr. were ground with laboratory mill SM-450 (MRC Laboratory Equipment Manufacturer, Holon, Israel) into a powder, and 25 g of this powder was mixed with 150 mL 70% methanol. The mixture was then placed in an ultrasonic bath (Sonorex Digitec DT 510 H-RC, Bandelin electronic GmbH & Co. KG, Berlin, Germany) for 30 min. After sonication, the sample was left for 48 h in a dark place at room temperature (22 °C). Following this period, the mixture was filtered using Whatman No. 1 filter paper and evaporated. A portion of 0.100 g of the crude extract was mixed with 10 mL demineralized water and stirred for 30 min. Subsequently, 10 mL of this mixture was mixed with 90 mL of 1 mM AgNO_3_ and the pH was adjusted from 4.9 to 7 using 0.2 M NaOH. This mixture was placed in a shaking bath (Memmert Water Bath WNB 22 with Memmert SV 1422 Shaking Device, Memmert GmbH + Co. KG, Nürnberg, Germany) at 80 °C until a color change was observed. The reaction mixture’s color changed from yellow to reddish brown (Figure S1A,B), signifying the reduction of silver ions to elemental silver. The resulting mixture was centrifuged for 45 min at 2000 rpm (Centrifuge Tehtnica Centric 322 A, Domel d.o.o., Slovenia) to separate AgNPs. The AgNPs were then re-dispersed in demineralized water and subjected to three additional washing steps via repeated centrifugations at 2000 rpm for 45 min to ensure the removal of any free bioactive compounds from *F. fomentarius*. After each round of centrifugation, the supernatant was analyzed using UV-Vis spectroscopy to confirm the absence of AgNO_3_. The resulting pellets were then dried at room temperature (22–24 °C) for 96 h. These thoroughly purified and dried samples were subsequently used for Powder X-ray Diffraction (PXRD), Transmission Electron Microscopy (TEM) analyses, and antibacterial testing. In this study, the optimal parameters for silver nanoparticle synthesis were determined to be 1 mM AgNO_3_, a temperature of 80 °C, a 1:9 extract to AgNO_3_ solution ratio, and a pH of 7.

### 3.3. Characterization of AgNPs

The UV-Vis spectra of the synthesized AgNPs were collected using a UV-1900 Shimadzu spectrophotometer (Shimadzu Corporation, Kyoto, Japan) over a spectral range of 350–700 nm with a 1 cm cuvette. The FT-IR spectra, ranging from 400 to 4000 cm^−1^, were acquired using an FT-IR 8400s Shimadzu spectrometer (Shimadzu Corporation, Kyoto, Japan). After FT-IR analysis, powder X-ray diffraction (PXRD) patterns were obtained using a Panalytical Aeris X-ray diffractometer (Malvern Panalytical Ltd., Malvern, UK). The Transmission Electron Microscopy (TEM) analysis was performed using JEOL JEM 1200EX II TEM (Jeol Ltd., Tokyo, Japan) at the Institute of Pharmaceutical Technology and Biopharmacy, Medical School, University of Pécs, Hungary. For TEM analysis, samples were placed on the 200 mesh Butvar B-98-coated copper grid (Micro to Nano Ltd., Haarlem, The Netherlands). The software ImageJ 1.54f was used to estimate the diameters of visualized AgNPs.

### 3.4. Antibacterial Activity of AgNPs

Using a modified broth microdilution method, a minimum inhibitory concentration (MIC) test was used to evaluate the antibacterial capabilities of silver nanoparticles [100] against four different bacterial strains: Gram-negative *Pseudomonas aeruginosa*, *Escherichia coli*, and Gram-positive *Bacillus subtilis* and *Staphylococcus aureus*. The bacterial strains in consideration were isolated from a range of clinical samples acquired from the Institute of Public Health Osijek, situated in Osijek-Baranja County, Croatia, and operated by the Microbiology Service. Polypropylene flat sterile 96-well microtiter plates (Kartell Labware, Noviglio (MI), Italy) were used for the broth microdilution assays. Bacterial subcultures (5 × 10^5^ CFU mL^−1^) in Mueller Hinton Broth (Cultimed, Barcelona, Spain) were added to dual serial diluted synthesized AgNPs, which were resuspended in methanol for this microdilution method. Each plate had wells with a bacterial subculture without AgNPs for growth control and wells with just broth and methanol for solvent (continuous phase) control. Additionally, we conducted an antibacterial assay for the initial methanolic extract of *Fomes fomentarius* L. Fr. itself. Under identical conditions, a co-assay was conducted using the antibiotic ciprofloxacin (Hospira, Hurley, Maidenhead, Berkshire, England, UK). Triphenyl tetrazolium chloride (VWR Chemicals, Leuven, Belgium) served as a bacterial growth indicator during an extra three-hour incubation period at 37 °C following a 24-h incubation period at the same temperature. The minimum inhibitory concentration (MIC) was determined as the lowest concentration of the AgNPs, obtained from triplicate analyses normalized against the solvent control.

## 4. Conclusions

This study successfully demonstrated the green synthesis of silver nanoparticles (AgNPs) using the methanolic extract of *Fomes fomentarius* L. Fr. The synthesized AgNPs were thoroughly characterized, with UV-Vis spectroscopy confirming their formation through a sharp absorption peak at 423 nm. FT-IR analysis identified the key biomolecules responsible for the reduction and stabilization of the nanoparticles, while PXRD analysis confirmed their crystalline nature, showing a face-centered cubic (fcc) structure with an average crystallite size of approximately 24 nm, in accordance with TEM analysis. The antibacterial efficacy of the synthesized AgNPs was evident, particularly against *S. aureus*, with the nanoparticles showing higher activity against gram-positive bacteria. This positions the AgNPs synthesized using *Fomes fomentarius* L. Fr. as a promising candidate for applications in antibacterial treatments, particularly in targeting resistant bacterial strains.

While this study provides significant insights into the potential of *Fomes fomentarius* L. Fr. in the synthesis of functional AgNPs, it also highlights the need for further research. The study’s limitations emphasize the necessity of employing additional characterization techniques such as Dynamic Light Scattering (DLS) and X-ray Photoelectron Spectroscopy (XPS) in future research. These techniques could offer a deeper understanding of the surface chemistry and stability of the nanoparticles, which are critical for their application in biomedical and biotechnological fields. Additionally, investigating the specific molecular mechanisms of interaction between the AgNPs and bacterial cells, including the generation of reactive oxygen species and the potential disruption of bacterial biofilms, continues to be an important topic for research.

In conclusion, this study not only introduces a novel approach to AgNP synthesis using *Fomes fomentarius* L. Fr. but also provides the basis for future research intended to enhance the characterization and application of these nanoparticles. The findings contribute to the expanding data on the green synthesis of nanoparticles, emphasizing the potential of mushrooms as a sustainable and effective source for nanoparticle production.

## Figures and Tables

**Table 1:** Minimum inhibitory concentrations (MIC) of methanolic extract of *Fomes fomentarius* L. Fr. and AgNPs synthesized using methanolic extract of *Fomes fomentarius* L. Fr. against *Escherichia coli, Pseudomonas aeruginosa, Bacillus subtilis*, and *Staphylococcus aureus* (µg mL^−1^).

	MIC (µg mL^−1^)			
	*B. subtilis*	*S. aureus*	*E. coli*	*P. aeruginosa*
*Fomes fomentarius* MetOH extract *	20.83	10.41	2.63	20.83
AgNPs	12.69	6.34	12.69	12.69
Ciprofloxacin	1.56	3.13	3.13	7.89

* Results expressed as mg mL^−1^.

## Data Availability

The data presented in this study are available on request from the corresponding author.

## Supplementary Materials

The following supporting information can be downloaded at: https://www.mdpi.com/article/10.3390/molecules29163961/s1, Figure S1: (A) *Fomes fomentarius* L. Fr. methanolic extract before and (B) after the addition of 1 mM AgNO_3_; Figure S2: Original unprocessed Transmission Electron Microscopy (TEM) image of silver nanoparticles (AgNPs) synthesized using methanolic extract of *Fomes fomentarius* L. Fr., obtained using a JEM 1200EX II Transmission Electron Microscope.
