# Supplementary figures and images for: Antibacterial Efficacy and Characterization of Silver Nanoparticles Synthesized via Methanolic Extract of Fomes fomentarius L. Fr
Source: Molecules. 2024 Aug 22;29(16):3961. doi: 10.3390/molecules29163961 (PMC11357466; doi:10.3390/molecules29163961)

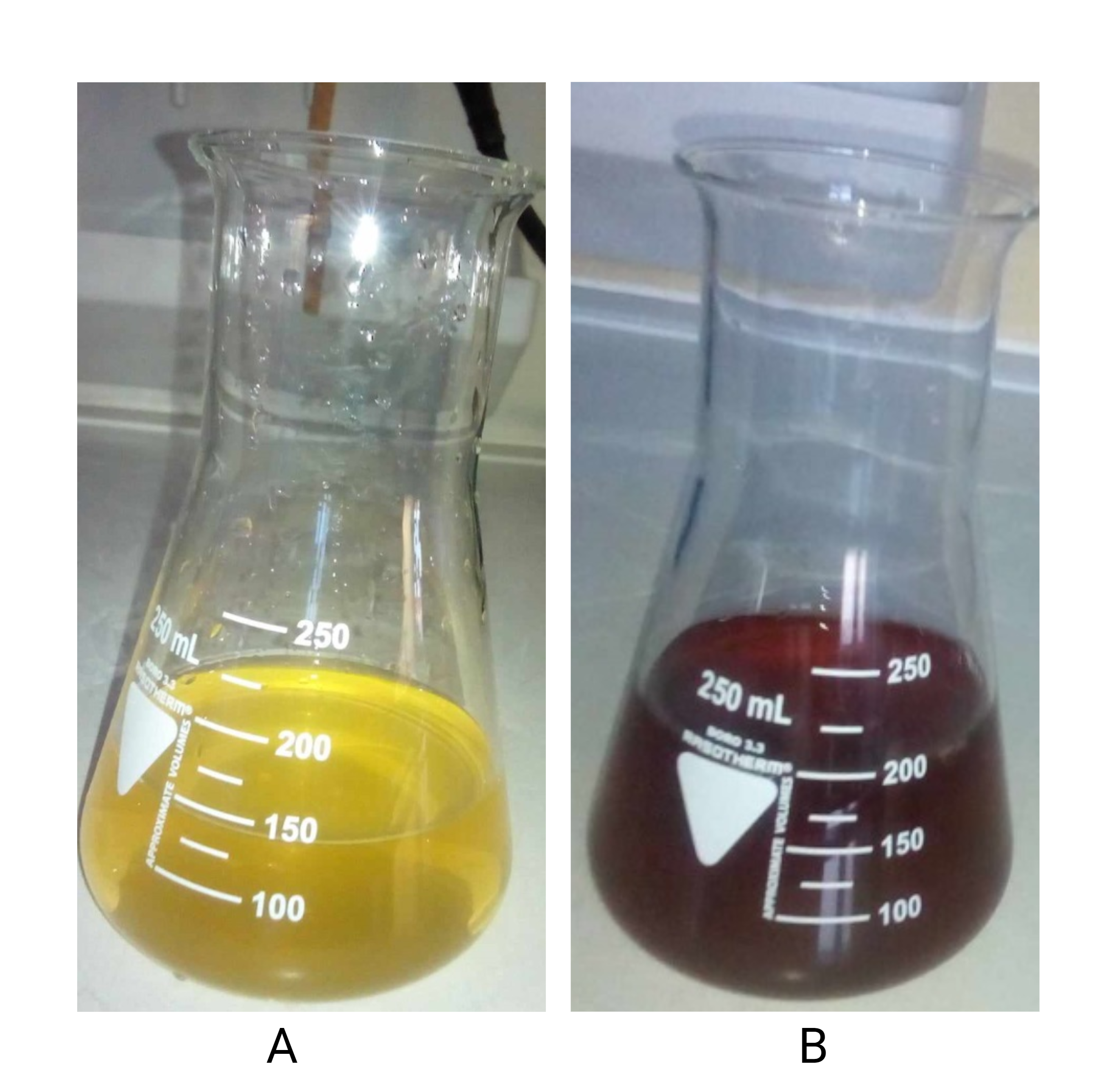

Supplement: Supplementary file 1 [file molecules-29-03961-s001.zip › Figure S1.png]

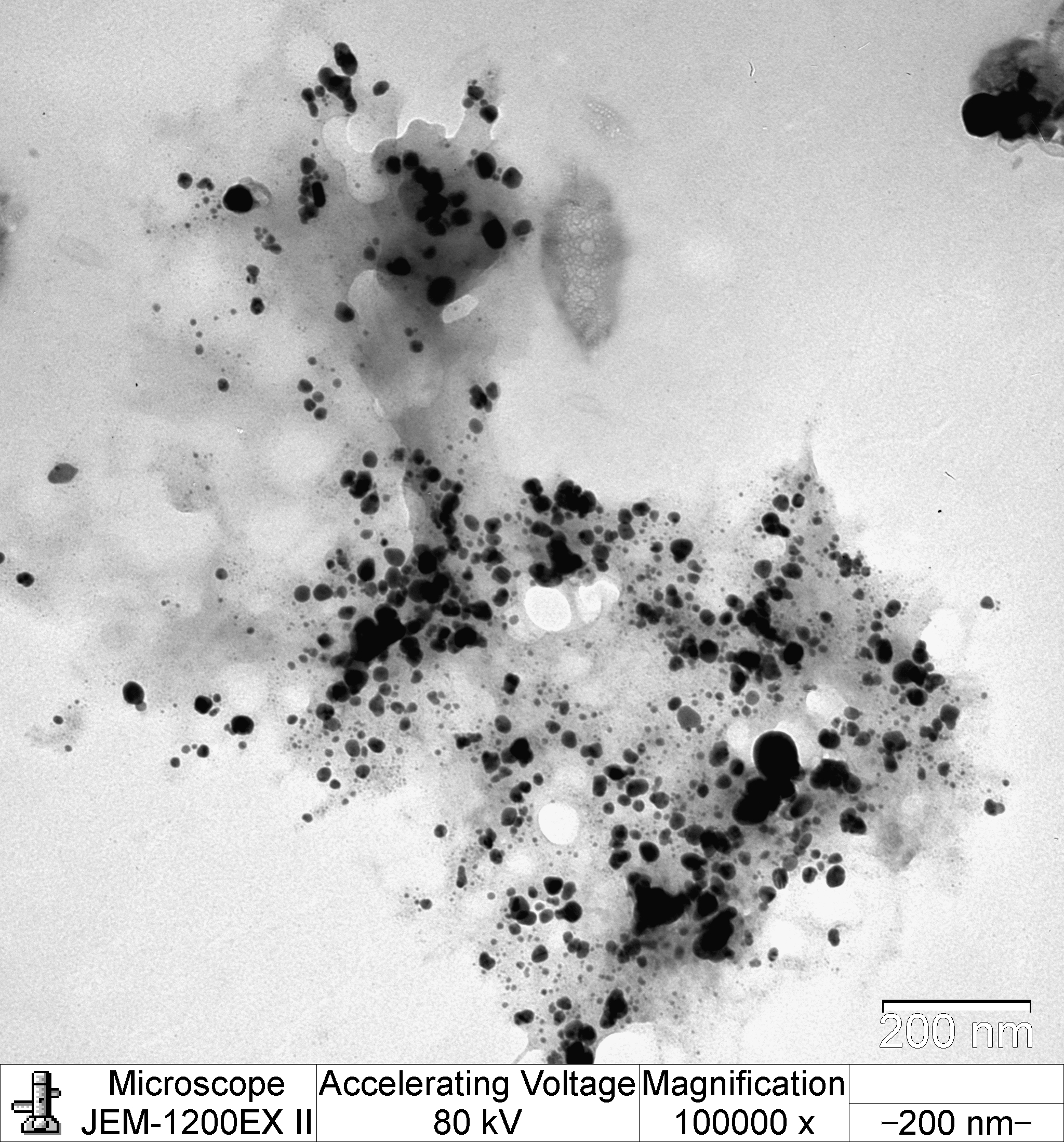

Supplement: Supplementary file 1 [file molecules-29-03961-s001.zip › Figure S2.png]
